# [3-(Dimethyl­amino)benzoato]triphenyl­tin(IV)

**DOI:** 10.1107/S1600536808036337

**Published:** 2008-11-13

**Authors:** Yip Foo Win, Siang Guan Teoh, Sie Tiong Ha, Reza Kia, Hoong-Kun Fun

**Affiliations:** a School of Chemical Sciences, Universiti Sains Malaysia, 11800 USM, Penang, Malaysia; b Universiti Tunku Abdul Rahman, Faculty of Engineering and Science, Jalan Genting Kelang, Setapak 53300, Kuala Lumpur, Malaysia; c X-ray Crystallography Unit, School of Physics, Universiti Sains Malaysia, 11800 USM, Penang, Malaysia

## Abstract

In the title compound, [Sn(C_6_H_5_)_3_(C_9_H_10_NO_2_)], the Sn atom is coordinated by three phenyl groups and a carboxyl­ate anion in a distorted tetra­hedral geometry. An intra­molecular C—H⋯O inter­action forms an *S*(7) ring motif. The dihedral angles between the benzoate group and the other three phenyl rings are 76.94 (8), 66.82 (8) and 42.34 (9)°. The crystal structure is further stabilized by inter­molecular C—H⋯π inter­actions.

## Related literature

For hydrogen-bond motifs, see Bernstein *et al.* (1995 ▶). For values of bond lengths, see Allen *et al.* (1987 ▶). For related literature on triorganotin(IV) complexes see, for example: Willem *et al.* (1997 ▶); Novelli *et al.* (1999 ▶); Gielen *et al.* (2000 ▶); Tian *et al.* (2005 ▶); Baul *et al.* (2001 ▶); Win *et al.* (2006 ▶, 2007*a* ▶,*b* ▶); Yeap & Teoh (2003 ▶).
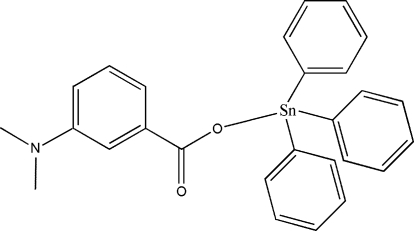


## Experimental

### 

#### Crystal data

[Sn(C_6_H_5_)_3_(C_9_H_10_NO_2_)]
*M* *_r_* = 514.17
Triclinic, 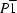

*a* = 9.1140 (2) Å
*b* = 10.0027 (2) Å
*c* = 14.5066 (4) Å
α = 100.925 (1)°
β = 103.106 (1)°
γ = 110.778 (1)°
*V* = 1150.13 (5) Å^3^
*Z* = 2
Mo *K*α radiation
μ = 1.13 mm^−1^
*T* = 100.0 (1) K
0.46 × 0.42 × 0.17 mm

#### Data collection

Bruker SMART APEXII CCD area-detector diffractometer
Absorption correction: multi-scan (**SADABS**; Bruker, 2005 ▶) *T*_min_ = 0.623, *T*_max_ = 0.830
18268 measured reflections
5259 independent reflections
5141 reflections with *I* > 2σ(*I*)
*R*_int_ = 0.017

#### Refinement

*R*[*F* ^2^ > 2σ(*F* ^2^)] = 0.017
*wR*(*F* ^2^) = 0.049
*S* = 1.08
5259 reflections
282 parameters
H-atom parameters constrained
Δρ_max_ = 0.53 e Å^−3^
Δρ_min_ = −0.56 e Å^−3^

### 

Data collection: *APEX2* (Bruker, 2005 ▶); cell refinement: *APEX2*; data reduction: *SAINT* (Bruker, 2005 ▶); program(s) used to solve structure: *SHELXTL* (Sheldrick, 2008 ▶); program(s) used to refine structure: *SHELXTL*; molecular graphics: *SHELXTL*; software used to prepare material for publication: *SHELXTL* and *PLATON* (Spek, 2003 ▶).

## Supplementary Material

Crystal structure: contains datablocks global, I. DOI: 10.1107/S1600536808036337/kp2195sup1.cif

Structure factors: contains datablocks I. DOI: 10.1107/S1600536808036337/kp2195Isup2.hkl

Additional supplementary materials:  crystallographic information; 3D view; checkCIF report

## Figures and Tables

**Table 1:** Selected bond lengths (Å)

Sn1—O1	2.0649 (11)
Sn1—C1	2.1239 (15)
Sn1—C13	2.1260 (14)
Sn1—C7	2.1290 (14)

**Table 2:** Hydrogen-bond geometry (Å, °)

*D*—H⋯*A*	*D*—H	H⋯*A*	*D*⋯*A*	*D*—H⋯*A*
C8—H8*A*⋯O2	0.93	2.43	3.126 (2)	132
C24—H24*A*⋯*Cg*1^i^	0.93	2.88	3.6772 (19)	144
C26—H26*B*⋯*Cg*2^ii^	0.96	2.74	3.672 (2)	164

Symmetry codes: (i) 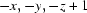; (ii) 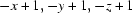.

**Fig. 1.**
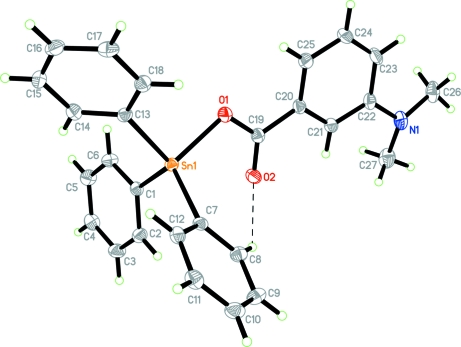
The molecular structure of the title compound with atom lables and the 50% probability ellipsoids for non-H atoms. Intramolecular hydrogen bonds is shown as dashed lines.

**Fig. 2.**
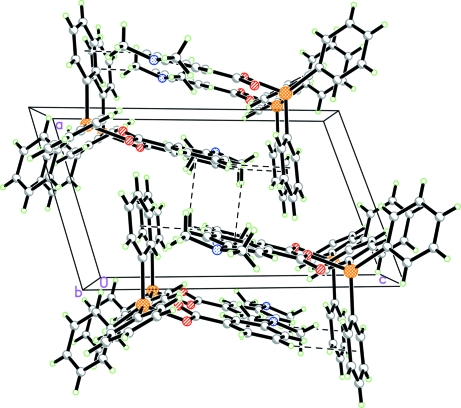
The crystal structure of (I), viewed down the b-axis. Intermolecular C—H···π interactions were shown as dashed lines.

## supplementary crystallographic information

### Comment

Triorganotin(IV) complexes are well known for their biological properties as well as industrial applications (Willem *et al.*, 1997; Novelli *et al.*, 1999; Gielen *et al.*, 2000; Tian *et al.*, 2005). Generally, triphenyltin(IV) carboxylate complexes are commonly found as monomeric structures with four-coordinated distorted tetrahedral or five-coordinated trigonal bipyramid geometries (Baul *et al.*, 2001; Yeap & Teoh, 2003; Win *et al.*, 2007*b*). In a recent study, the coordination geometry of (3,5-dinitrobenzoato)triphenyltin(IV) is found to be distorted tetrahedral due to the long range interaction of the carboxylate anion coordinated to the Sn moiety in an isobidentate fashion (Win *et al.*, 2006). In addition, triphenyltin(IV) carboxylates are also able to form polymeric structures (Tian *et al.*, 2005; Win *et al.*, 2007a). In the polymeric system, the carboxylate anions act as bridging bidentate ligands in the bonding to the neighbouring tin(IV) resulting in a polymeric structure with the tin atom exhibiting trigonal bipyramid geometry as shown in the complex, *catena*-poly[[triphenyltin(IV)–2,4-dinitrobenzoato] (Win *et al.*, 2007a). Based on the crystallographic structural study, the title complex [3-(dimethylamino)benzoato]triphenyltin(IV) has a monomeric four-coordinated distorted tetrahedral structure which is similar to that found for [4-(diethylamino)benzoato-κO]triphenyltin(IV) (Win *et al.*, 2007*b*).

The bond lengths (Allen *et al.*, 1987) and angles in the molecule (I, Fig. 1, Table 1) are within normal ranges. The Sn atom is coordinated by the three phenyl groups and a carboxylate anion in a distorted tetrahedral geometry. An intramolecular hydrogen bond C—H···O forms a seven-membered ring, characterized as *S(7)* motif (Bernstein *et al.*, 1995). The dihedral angles between the phenyl-carboxylate group and the other three phenyl rings are 76.94 (8), 66.82 (8), and 42.34 (9)°, respectively. The crystal structure (Fig. 2), is further stabilized by intermolecular C—H···π (*x* 2) (Table 2) interactions.

### Experimental

The complex [3-(dimethylamino)benzoato]triphenyltin(IV) was obtained by heating under reflux a 1:1 molar mixture of triphenyltin(IV) hydroxide (1.10 g, 3 mmol) and 3-(dimethylamino)benzoic acid (0.50 g, 3 mmol) in acetonitrile (50 ml) for an hour. The clear brown solution was isolated by filtration and kept in a bottle. After eight days, brown crystals (1.01 g, 65.7% yield) were collected. Melting point: 413.2–414.5 K. Analysis found for C_27_H_25_NO_2_Sn: C, 63.05; H, 4.91; N, 2.67; Sn, 23.00%. Calculated found for C_27_H_25_NO_2_Sn: C, 63.07; H, 4.90; N, 2.72; Sn, 23.08%. FTIR as KBr disc (cm_-1_): υ (C—H) aromatic 3065, 3051, 3026; υ (C—H) saturated 2989, 2908, 2810; υ (COO)_as_ 1625, υ (COO)_s_ 1322, υ (C—N) 1227, υ (Sn—O) 445. ^1^H-NMR: δ: phenyl protons 7.42–7.49 (9*H*, m); 7.79–7.81 (6*H*, m); benzene 6.86–6.88 (1*H*, dd); 7.24–7.28 (1*H*, t); 7.51–7.53 (2*H*, d); N-(CH3)2 2.95 (6*H*, s) p.p.m.. ^13^C-NMR: δ: phenyl carbons C*_ipso_* 139.01 (648.9 Hz), C*_ortho_* 137.36 (47.9 Hz), C*_meta_* 129.31 (63.2 Hz), C*_para_* 130.28; benzene 114.84, 117.18, 119.31, 129.57, 134.59, 150.85; N-(CH_3_)_2_ 41.04; COO 174.05 p.p.m.. ^119^Sn-NMR: υ: -114.19 p.p.m..

### Refinement

All of the hydrogen atoms were positioned geometrically and refined using a riding model with C—H = 0.93 Å for aromatic H and 0.96 Å for methyl H atoms. A rotating group model was used for the methyl groups.

### Crystal data

**Table:**

[Sn(C_6_H_5_)_3_(C_9_H_10_NO_2_)]	*Z* = 2
*M**_r_* = 514.17	*F*_000_ = 520
Triclinic, *P*1	*D*_x_ = 1.485 Mg m^−^^3^
Hall symbol: -P 1	Mo *K*α radiation λ = 0.71073 Å
*a* = 9.1140 (2) Å	Cell parameters from 9986 reflections
*b* = 10.0027 (2) Å	θ = 2.5–31.2º
*c* = 14.5066 (4) Å	µ = 1.13 mm^−^^1^
α = 100.925 (1)º	*T* = 100.0 (1) K
β = 103.106 (1)º	Block, colourless
γ = 110.778 (1)º	0.46 × 0.42 × 0.17 mm
*V* = 1150.13 (5) Å^3^	

### Data collection

**Table:**

Bruker SMART APEXII CCD area-detector diffractometer	5259 independent reflections
Radiation source: fine-focus sealed tube	5141 reflections with *I* > 2σ(*I*)
Monochromator: graphite	*R*_int_ = 0.017
*T* = 100.0(1) K	θ_max_ = 27.5º
φ and ω scans	θ_min_ = 2.3º
Absorption correction: multi-scan(*SADABS*; Bruker, 2005)	*h* = −11→11
*T*_min_ = 0.623, *T*_max_ = 0.830	*k* = −12→12
18268 measured reflections	*l* = −18→18

### Refinement

**Table:**

Refinement on *F*^2^	Secondary atom site location: difference Fourier map
Least-squares matrix: full	Hydrogen site location: inferred from neighbouring sites
*R*[*F*^2^ > 2σ(*F*^2^)] = 0.017	H-atom parameters constrained
*wR*(*F*^2^) = 0.049	*w* = 1/[σ^2^(*F*_o_^2^) + (0.0269*P*)^2^ + 0.592*P*] where *P* = (*F*_o_^2^ + 2*F*_c_^2^)/3
*S* = 1.08	(Δ/σ)_max_ = 0.001
5259 reflections	Δρ_max_ = 0.53 e Å^−^^3^
282 parameters	Δρ_min_ = −0.56 e Å^−^^3^
Primary atom site location: structure-invariant direct methods	Extinction correction: none

### Special details

**Table:**

Experimental. The low-temperature data was collected with the Oxford Cyrosystem Cobra low-temperature attachment.
Geometry. All e.s.d.'s (except the e.s.d. in the dihedral angle between two l.s. planes) are estimated using the full covariance matrix. The cell e.s.d.'s are taken into account individually in the estimation of e.s.d.'s in distances, angles and torsion angles; correlations between e.s.d.'s in cell parameters are only used when they are defined by crystal symmetry. An approximate (isotropic) treatment of cell e.s.d.'s is used for estimating e.s.d.'s involving l.s. planes.
Refinement. Refinement of *F*^2^ against ALL reflections. The weighted *R*-factor *wR* and goodness of fit *S* are based on *F*^2^, conventional *R*-factors *R* are based on *F*, with *F* set to zero for negative *F*^2^. The threshold expression of *F*^2^ > σ(*F*^2^) is used only for calculating *R*-factors(gt) *etc*. and is not relevant to the choice of reflections for refinement. *R*-factors based on *F*^2^ are statistically about twice as large as those based on *F*, and *R*- factors based on ALL data will be even larger.

### Fractional atomic coordinates and isotropic or equivalent isotropic displacement parameters (Å^2^)

**Table:**

	*x*	*y*	*z*	*U*_iso_*/*U*_eq_	
Sn1	0.091614 (11)	0.100499 (9)	0.838745 (6)	0.01429 (4)	
O1	0.18084 (14)	0.11930 (12)	0.72088 (8)	0.0197 (2)	
O2	0.11484 (14)	0.31350 (12)	0.73771 (8)	0.0204 (2)	
N1	0.22892 (18)	0.51484 (16)	0.45101 (10)	0.0254 (3)	
C1	0.22847 (18)	0.28442 (15)	0.96940 (10)	0.0159 (3)	
C2	0.14887 (19)	0.36829 (17)	1.00604 (11)	0.0207 (3)	
H2A	0.0400	0.3462	0.9710	0.025*	
C3	0.2303 (2)	0.48439 (18)	1.09426 (12)	0.0244 (3)	
H3A	0.1762	0.5399	1.1177	0.029*	
C4	0.3920 (2)	0.51738 (17)	1.14722 (12)	0.0229 (3)	
H4A	0.4465	0.5948	1.2064	0.028*	
C5	0.4729 (2)	0.43461 (18)	1.11195 (12)	0.0232 (3)	
H5A	0.5816	0.4570	1.1474	0.028*	
C6	0.39153 (19)	0.31856 (17)	1.02389 (12)	0.0205 (3)	
H6A	0.4459	0.2631	1.0010	0.025*	
C7	−0.16663 (18)	0.04727 (16)	0.79258 (10)	0.0158 (3)	
C8	−0.23183 (19)	0.14256 (16)	0.75791 (11)	0.0194 (3)	
H8A	−0.1615	0.2315	0.7516	0.023*	
C9	−0.4012 (2)	0.10510 (18)	0.73291 (12)	0.0233 (3)	
H9A	−0.4435	0.1683	0.7089	0.028*	
C10	−0.5073 (2)	−0.02620 (19)	0.74368 (12)	0.0240 (3)	
H10A	−0.6201	−0.0501	0.7280	0.029*	
C11	−0.4443 (2)	−0.12170 (18)	0.77797 (12)	0.0230 (3)	
H11A	−0.5151	−0.2100	0.7849	0.028*	
C12	−0.27509 (19)	−0.08543 (17)	0.80205 (11)	0.0188 (3)	
H12A	−0.2338	−0.1501	0.8247	0.023*	
C13	0.13625 (18)	−0.08981 (16)	0.85493 (11)	0.0164 (3)	
C14	0.2177 (2)	−0.09087 (18)	0.94868 (12)	0.0215 (3)	
H14A	0.2560	−0.0066	1.0028	0.026*	
C15	0.2422 (2)	−0.21653 (19)	0.96204 (12)	0.0251 (3)	
H15A	0.2965	−0.2160	1.0248	0.030*	
C16	0.1856 (2)	−0.34238 (18)	0.88169 (13)	0.0245 (3)	
H16A	0.1999	−0.4271	0.8907	0.029*	
C17	0.1073 (2)	−0.34218 (17)	0.78765 (13)	0.0225 (3)	
H17A	0.0713	−0.4260	0.7336	0.027*	
C18	0.08268 (18)	−0.21649 (17)	0.77429 (11)	0.0193 (3)	
H18A	0.0302	−0.2169	0.7112	0.023*	
C19	0.16464 (18)	0.23129 (16)	0.69218 (11)	0.0168 (3)	
C20	0.20934 (18)	0.25094 (16)	0.60128 (10)	0.0172 (3)	
C21	0.20685 (18)	0.37521 (16)	0.57167 (11)	0.0184 (3)	
H21A	0.1814	0.4443	0.6098	0.022*	
C22	0.24224 (19)	0.39733 (17)	0.48506 (11)	0.0199 (3)	
C23	0.2867 (2)	0.29256 (19)	0.43204 (11)	0.0239 (3)	
H23A	0.3153	0.3064	0.3758	0.029*	
C24	0.2888 (2)	0.16944 (19)	0.46203 (12)	0.0246 (3)	
H24A	0.3171	0.1014	0.4251	0.030*	
C25	0.24929 (19)	0.14608 (17)	0.54641 (11)	0.0207 (3)	
H25A	0.2494	0.0627	0.5659	0.025*	
C26	0.2998 (2)	0.5495 (2)	0.37333 (13)	0.0306 (4)	
H26A	0.2477	0.4636	0.3157	0.046*	
H26B	0.4166	0.5755	0.3964	0.046*	
H26C	0.2817	0.6321	0.3568	0.046*	
C27	0.2286 (2)	0.64104 (19)	0.52007 (14)	0.0318 (4)	
H27A	0.1372	0.6063	0.5449	0.048*	
H27B	0.2177	0.7127	0.4865	0.048*	
H27C	0.3305	0.6873	0.5743	0.048*	

### Atomic displacement parameters (Å^2^)

**Table:**

	*U*^11^	*U*^22^	*U*^33^	*U*^12^	*U*^13^	*U*^23^
Sn1	0.01477 (6)	0.01395 (6)	0.01457 (6)	0.00679 (4)	0.00447 (4)	0.00385 (4)
O1	0.0237 (5)	0.0211 (5)	0.0189 (5)	0.0116 (4)	0.0093 (4)	0.0091 (4)
O2	0.0243 (6)	0.0207 (5)	0.0194 (5)	0.0105 (4)	0.0108 (4)	0.0065 (4)
N1	0.0309 (7)	0.0256 (7)	0.0197 (6)	0.0100 (6)	0.0072 (6)	0.0110 (5)
C1	0.0177 (7)	0.0146 (6)	0.0149 (6)	0.0058 (5)	0.0057 (5)	0.0047 (5)
C2	0.0187 (7)	0.0234 (7)	0.0200 (7)	0.0105 (6)	0.0043 (6)	0.0057 (6)
C3	0.0273 (8)	0.0245 (8)	0.0235 (8)	0.0143 (7)	0.0097 (7)	0.0029 (6)
C4	0.0250 (8)	0.0190 (7)	0.0189 (7)	0.0055 (6)	0.0057 (6)	0.0018 (6)
C5	0.0176 (7)	0.0235 (7)	0.0231 (8)	0.0060 (6)	0.0035 (6)	0.0042 (6)
C6	0.0180 (7)	0.0206 (7)	0.0231 (7)	0.0093 (6)	0.0067 (6)	0.0041 (6)
C7	0.0155 (6)	0.0180 (6)	0.0125 (6)	0.0076 (5)	0.0041 (5)	0.0005 (5)
C8	0.0193 (7)	0.0170 (6)	0.0205 (7)	0.0079 (6)	0.0059 (6)	0.0028 (5)
C9	0.0210 (7)	0.0223 (7)	0.0268 (8)	0.0122 (6)	0.0050 (6)	0.0045 (6)
C10	0.0162 (7)	0.0289 (8)	0.0228 (8)	0.0082 (6)	0.0055 (6)	0.0023 (6)
C11	0.0200 (7)	0.0239 (7)	0.0205 (7)	0.0044 (6)	0.0067 (6)	0.0054 (6)
C12	0.0203 (7)	0.0198 (7)	0.0149 (7)	0.0077 (6)	0.0045 (6)	0.0042 (5)
C13	0.0151 (6)	0.0169 (6)	0.0198 (7)	0.0076 (5)	0.0072 (5)	0.0072 (5)
C14	0.0257 (8)	0.0234 (7)	0.0180 (7)	0.0120 (6)	0.0084 (6)	0.0064 (6)
C15	0.0289 (8)	0.0341 (9)	0.0236 (8)	0.0196 (7)	0.0119 (7)	0.0163 (7)
C16	0.0256 (8)	0.0245 (8)	0.0366 (9)	0.0162 (6)	0.0178 (7)	0.0167 (7)
C17	0.0217 (7)	0.0175 (7)	0.0296 (8)	0.0088 (6)	0.0115 (6)	0.0047 (6)
C18	0.0175 (7)	0.0195 (7)	0.0192 (7)	0.0073 (6)	0.0045 (6)	0.0050 (6)
C19	0.0147 (6)	0.0174 (6)	0.0152 (6)	0.0048 (5)	0.0031 (5)	0.0041 (5)
C20	0.0161 (7)	0.0199 (7)	0.0135 (6)	0.0063 (5)	0.0035 (5)	0.0041 (5)
C21	0.0186 (7)	0.0191 (7)	0.0158 (7)	0.0075 (5)	0.0044 (5)	0.0038 (5)
C22	0.0183 (7)	0.0223 (7)	0.0146 (7)	0.0053 (6)	0.0022 (5)	0.0055 (6)
C23	0.0256 (8)	0.0325 (8)	0.0127 (7)	0.0114 (7)	0.0068 (6)	0.0056 (6)
C24	0.0280 (8)	0.0300 (8)	0.0156 (7)	0.0150 (7)	0.0065 (6)	0.0009 (6)
C25	0.0228 (7)	0.0211 (7)	0.0170 (7)	0.0101 (6)	0.0043 (6)	0.0035 (6)
C26	0.0266 (8)	0.0360 (9)	0.0241 (8)	0.0052 (7)	0.0054 (7)	0.0164 (7)
C27	0.0408 (10)	0.0243 (8)	0.0311 (9)	0.0139 (7)	0.0091 (8)	0.0125 (7)

### Geometric parameters (Å, °)

**Table:**

Sn1—O1	2.0649 (11)	C12—H12A	0.9300
Sn1—C1	2.1239 (15)	C13—C18	1.397 (2)
Sn1—C13	2.1260 (14)	C13—C14	1.398 (2)
Sn1—C7	2.1290 (14)	C14—C15	1.393 (2)
O1—C19	1.3101 (17)	C14—H14A	0.9300
O2—C19	1.2303 (19)	C15—C16	1.385 (2)
N1—C22	1.391 (2)	C15—H15A	0.9300
N1—C27	1.457 (2)	C16—C17	1.389 (2)
N1—C26	1.458 (2)	C16—H16A	0.9300
C1—C2	1.397 (2)	C17—C18	1.393 (2)
C1—C6	1.398 (2)	C17—H17A	0.9300
C2—C3	1.390 (2)	C18—H18A	0.9300
C2—H2A	0.9300	C19—C20	1.493 (2)
C3—C4	1.384 (2)	C20—C21	1.396 (2)
C3—H3A	0.9300	C20—C25	1.397 (2)
C4—C5	1.391 (2)	C21—C22	1.404 (2)
C4—H4A	0.9300	C21—H21A	0.9300
C5—C6	1.388 (2)	C22—C23	1.411 (2)
C5—H5A	0.9300	C23—C24	1.386 (2)
C6—H6A	0.9300	C23—H23A	0.9300
C7—C12	1.399 (2)	C24—C25	1.390 (2)
C7—C8	1.401 (2)	C24—H24A	0.9300
C8—C9	1.393 (2)	C25—H25A	0.9300
C8—H8A	0.9300	C26—H26A	0.9600
C9—C10	1.389 (2)	C26—H26B	0.9600
C9—H9A	0.9300	C26—H26C	0.9600
C10—C11	1.389 (2)	C27—H27A	0.9600
C10—H10A	0.9300	C27—H27B	0.9600
C11—C12	1.394 (2)	C27—H27C	0.9600
C11—H11A	0.9300		
			
O1—Sn1—C1	114.69 (5)	C15—C14—C13	120.83 (15)
O1—Sn1—C13	95.46 (5)	C15—C14—H14A	119.6
C1—Sn1—C13	110.92 (6)	C13—C14—H14A	119.6
O1—Sn1—C7	109.89 (5)	C16—C15—C14	119.87 (15)
C1—Sn1—C7	113.28 (6)	C16—C15—H15A	120.1
C13—Sn1—C7	111.31 (5)	C14—C15—H15A	120.1
C19—O1—Sn1	109.13 (9)	C15—C16—C17	120.07 (14)
C22—N1—C27	118.52 (13)	C15—C16—H16A	120.0
C22—N1—C26	118.12 (15)	C17—C16—H16A	120.0
C27—N1—C26	115.85 (14)	C16—C17—C18	120.06 (15)
C2—C1—C6	118.61 (14)	C16—C17—H17A	120.0
C2—C1—Sn1	118.67 (11)	C18—C17—H17A	120.0
C6—C1—Sn1	122.57 (11)	C17—C18—C13	120.51 (14)
C3—C2—C1	120.82 (14)	C17—C18—H18A	119.7
C3—C2—H2A	119.6	C13—C18—H18A	119.7
C1—C2—H2A	119.6	O2—C19—O1	121.43 (13)
C4—C3—C2	119.94 (15)	O2—C19—C20	122.89 (13)
C4—C3—H3A	120.0	O1—C19—C20	115.68 (13)
C2—C3—H3A	120.0	C21—C20—C25	121.04 (14)
C3—C4—C5	119.97 (15)	C21—C20—C19	118.15 (13)
C3—C4—H4A	120.0	C25—C20—C19	120.80 (13)
C5—C4—H4A	120.0	C20—C21—C22	120.90 (14)
C6—C5—C4	120.12 (15)	C20—C21—H21A	119.6
C6—C5—H5A	119.9	C22—C21—H21A	119.6
C4—C5—H5A	119.9	N1—C22—C21	121.46 (15)
C5—C6—C1	120.54 (14)	N1—C22—C23	121.24 (14)
C5—C6—H6A	119.7	C21—C22—C23	117.27 (14)
C1—C6—H6A	119.7	C24—C23—C22	121.36 (14)
C12—C7—C8	118.59 (13)	C24—C23—H23A	119.3
C12—C7—Sn1	118.36 (10)	C22—C23—H23A	119.3
C8—C7—Sn1	122.97 (11)	C23—C24—C25	121.01 (15)
C9—C8—C7	120.51 (14)	C23—C24—H24A	119.5
C9—C8—H8A	119.7	C25—C24—H24A	119.5
C7—C8—H8A	119.7	C24—C25—C20	118.36 (14)
C10—C9—C8	120.31 (15)	C24—C25—H25A	120.8
C10—C9—H9A	119.8	C20—C25—H25A	120.8
C8—C9—H9A	119.8	N1—C26—H26A	109.5
C9—C10—C11	119.76 (15)	N1—C26—H26B	109.5
C9—C10—H10A	120.1	H26A—C26—H26B	109.5
C11—C10—H10A	120.1	N1—C26—H26C	109.5
C10—C11—C12	120.11 (15)	H26A—C26—H26C	109.5
C10—C11—H11A	119.9	H26B—C26—H26C	109.5
C12—C11—H11A	119.9	N1—C27—H27A	109.5
C11—C12—C7	120.71 (14)	N1—C27—H27B	109.5
C11—C12—H12A	119.6	H27A—C27—H27B	109.5
C7—C12—H12A	119.6	N1—C27—H27C	109.5
C18—C13—C14	118.64 (13)	H27A—C27—H27C	109.5
C18—C13—Sn1	121.68 (11)	H27B—C27—H27C	109.5
C14—C13—Sn1	119.66 (11)		
			
C1—Sn1—O1—C19	−65.43 (10)	C7—Sn1—C13—C18	63.64 (13)
C13—Sn1—O1—C19	178.55 (10)	O1—Sn1—C13—C14	131.33 (12)
C7—Sn1—O1—C19	63.54 (10)	C1—Sn1—C13—C14	12.27 (13)
O1—Sn1—C1—C2	118.11 (11)	C7—Sn1—C13—C14	−114.83 (12)
C13—Sn1—C1—C2	−135.17 (11)	C18—C13—C14—C15	−1.3 (2)
C7—Sn1—C1—C2	−9.16 (13)	Sn1—C13—C14—C15	177.23 (12)
O1—Sn1—C1—C6	−66.39 (13)	C13—C14—C15—C16	0.1 (2)
C13—Sn1—C1—C6	40.33 (13)	C14—C15—C16—C17	1.2 (2)
C7—Sn1—C1—C6	166.35 (11)	C15—C16—C17—C18	−1.3 (2)
C6—C1—C2—C3	0.7 (2)	C16—C17—C18—C13	0.0 (2)
Sn1—C1—C2—C3	176.37 (12)	C14—C13—C18—C17	1.3 (2)
C1—C2—C3—C4	−0.4 (2)	Sn1—C13—C18—C17	−177.23 (11)
C2—C3—C4—C5	0.2 (2)	Sn1—O1—C19—O2	5.06 (17)
C3—C4—C5—C6	−0.2 (2)	Sn1—O1—C19—C20	−174.58 (10)
C4—C5—C6—C1	0.5 (2)	O2—C19—C20—C21	5.3 (2)
C2—C1—C6—C5	−0.7 (2)	O1—C19—C20—C21	−175.11 (13)
Sn1—C1—C6—C5	−176.25 (11)	O2—C19—C20—C25	−173.49 (14)
O1—Sn1—C7—C12	120.58 (11)	O1—C19—C20—C25	6.1 (2)
C1—Sn1—C7—C12	−109.69 (11)	C25—C20—C21—C22	0.9 (2)
C13—Sn1—C7—C12	16.12 (13)	C19—C20—C21—C22	−177.86 (13)
O1—Sn1—C7—C8	−62.74 (13)	C27—N1—C22—C21	18.9 (2)
C1—Sn1—C7—C8	67.00 (13)	C26—N1—C22—C21	167.49 (15)
C13—Sn1—C7—C8	−167.20 (12)	C27—N1—C22—C23	−163.14 (16)
C12—C7—C8—C9	−0.4 (2)	C26—N1—C22—C23	−14.6 (2)
Sn1—C7—C8—C9	−177.08 (12)	C20—C21—C22—N1	175.44 (14)
C7—C8—C9—C10	1.1 (2)	C20—C21—C22—C23	−2.5 (2)
C8—C9—C10—C11	−1.1 (2)	N1—C22—C23—C24	−175.42 (15)
C9—C10—C11—C12	0.4 (2)	C21—C22—C23—C24	2.6 (2)
C10—C11—C12—C7	0.3 (2)	C22—C23—C24—C25	−0.9 (3)
C8—C7—C12—C11	−0.3 (2)	C23—C24—C25—C20	−0.8 (2)
Sn1—C7—C12—C11	176.53 (11)	C21—C20—C25—C24	0.9 (2)
O1—Sn1—C13—C18	−50.20 (12)	C19—C20—C25—C24	179.56 (14)
C1—Sn1—C13—C18	−169.26 (11)		

### Hydrogen-bond geometry (Å, °)

**Table:**

*D*—H···*A*	*D*—H	H···*A*	*D*···*A*	*D*—H···*A*
C8—H8A···O2	0.93	2.43	3.126 (2)	132
C24—H24A···Cg1^i^	0.93	2.88	3.6772 (19)	144
C26—H26B···Cg2^ii^	0.96	2.74	3.672 (2)	164

Symmetry codes: (i) −*x*, −*y*, −*z*+1; (ii) −*x*+1, −*y*+1, −*z*+1.
